# Water Diffusion and Uptake in Injectable ETTMP/PEGDA Hydrogels

**DOI:** 10.1021/acs.jpcb.3c00861

**Published:** 2023-05-26

**Authors:** Paige N. Rockwell, James E. Maneval, Brandon M. Vogel, Erin L. Jablonski

**Affiliations:** Department of Chemical Engineering, Bucknell University, Lewisburg, Pennsylvania 17837, United States

## Abstract

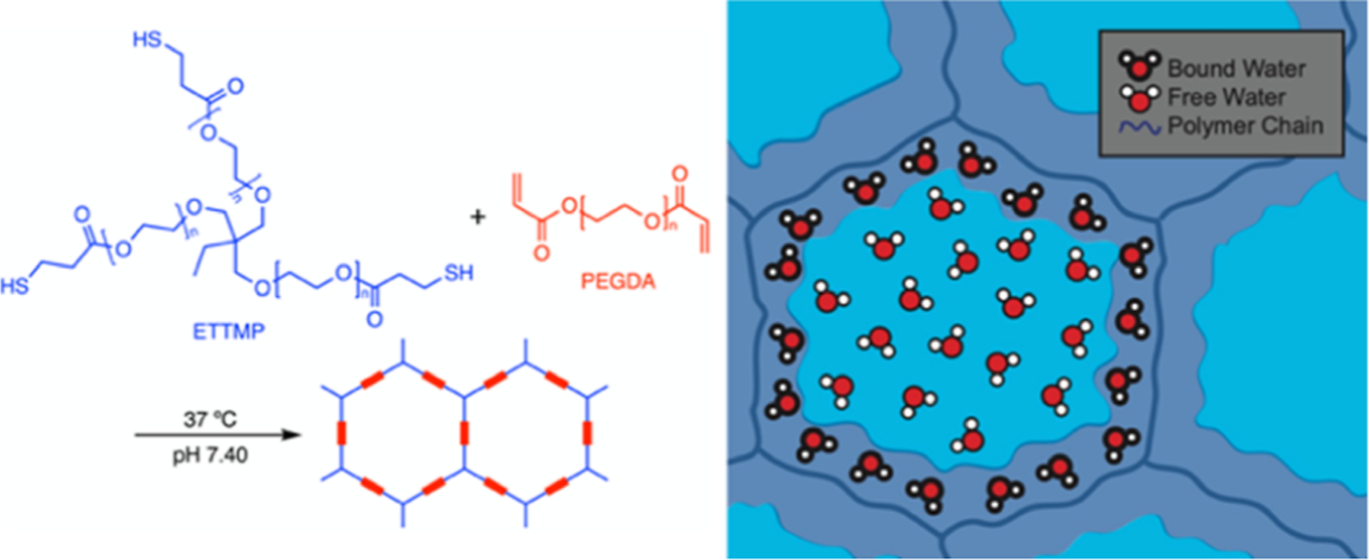

Differential scanning calorimetry (DSC) and pulsed field gradient spin echo nuclear magnetic resonance (PFGSE NMR) were used to characterize water in hydrogels of ethoxylated trimethylolpropane tri-3-mercaptopropionate (ETTMP) and poly(ethylene glycol) diacrylate (PEGDA). Freezable and nonfreezable water were quantified using DSC; water diffusion coefficients were measured using PFGSE NMR. No freezable water (free or intermediate) was detected from DSC for hydrogels of 0.68 and greater polymer mass fractions. Water diffusion coefficients, from NMR, decreased with increasing polymer content and were assumed to be weighted averages of free and bound water contributions. Both techniques showed decreasing ratios of bound or nonfreezable water mass per polymer mass with increasing polymer content. Swelling studies were used to quantify the equilibrium water content (EWC) to determine which compositions would swell or deswell when placed in the body. At 30 and 37 °C, fully cured, non-degraded ETTMP/PEGDA hydrogels at polymer mass fractions of 0.25 and 0.375, respectively, were shown to be at EWC.

## 1 Introduction

**Scheme 1:**
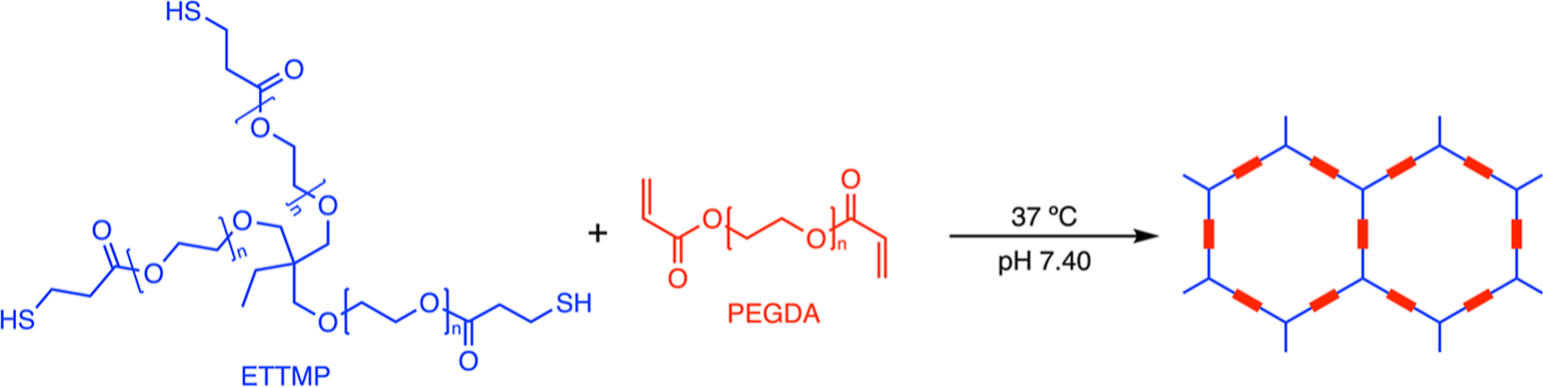
Thiol-Ene Michael Addition of ETTMP and PEGDA to Form a Hydrogel Network

Injectable, degradable hydrogels are of particular interest for use in local and controlled drug delivery applications because they can be administered once and allow for sustained release of therapy locally in vivo. Previous research has shown that the ethoxylated trimethylolpropane tri-3-mercaptopropionate (ETTMP) and poly(ethylene glycol) diacrylate (PEGDA) hydrogel system (Scheme 1) shows promise for potential use in drug delivery because of the commercial availability of ETTMP, quick in situ curing, reasonable degradation times, and potential for non-swelling behavior.^1−3^ Many hydrogel formulations exhibit swelling that can put pressure on nearby tissues and cause damage if placed in the body, so it is critical to understand the hydrogel swelling behavior to avoid unwanted side effects due to significant volume change. The desired hydrogel implant to be used in local controlled drug delivery will maintain volume and stay in place for much of the duration of drug release. Critical to understanding how the hydrogel matrix will perform in a drug delivery application is how water content within the hydrogel will change when placed in the body. Hydrogels synthesized from ETTMP/PEGDA have been shown to swell or shrink to different extents depending on temperature.^2^ Other hydrogels that exhibit this deswelling behavior have been reported by Zhou et al.^4^ The addition of Fe ions into calcium alginate/polyacrylamide hydrogels to form secondary ionic cross-linking led to areas of the hydrogel with an increased elastic modulus. This allowed for shape morphing of the hydrogel, due to areas with different swelling behaviors.^4^

Water in hydrogels exists in multiple physical forms influenced by the interactions between water molecules and constituent polymer chains. Bound water is tightly associated with polymer chains, and free water exists within network openings.^4^ There also exist intermediate populations of water that can be loosely bound to polymer chains or to tightly bound water. Nishida et al. recently summarized the interfacial roles of water with polymers, biomolecules, and inorganic materials and the impact of the interfacial water on the adsorption of biomolecules onto these surfaces. They identified three populations of water interacting with materials: free water, intermediate water, and nonfreezing water. Using this lens, they comprehensively review previous studies that classify water, the methods used to characterize the water populations, and the motion of the water (thermodynamics, structure, and dynamics). The techniques that have been used to classify water are differential scanning calorimetry (DSC), nuclear magnetic resonance (NMR), attenuated total reflection-Fourier transform infrared spectroscopy, dielectric relaxation spectroscopy, atomic force microscopy, X-ray analysis, small-angle neutron scattering, surface plasmon resonance spectroscopy, NS, terahertz (THz) spectroscopy, and MD simulations.^5^

Dargaville and Hutmacher offer further evidence that the properties of water bound to macromolecules measurably differ from those of free water.^6^ They present the nomenclature often found in the literature to describe various states of water: “hydration water, associated water, bound versus free water, fast versus slow water, and freezable versus nonfreezable water.” In hydrogels, water is described as existing in one of three states: bound, intermediate, and free. Free water behaves as bulk water in terms of freezing and melting. Intermediate water is understood as forming the secondary hydration shell, through hydrogen bonding to water molecules that are bound to the macromolecular chain.^6^

The present study distinguishes between freezable and nonfreezable water as determined by DSC measurement. According to Dargaville and Hutmacher, DSC can be used to quantify the relative populations of bound, intermediate, and free water in hydrogels and is appropriately complemented by water behavior determined via NMR. Notably, the authors appreciate the value of using phosphate-buffered saline (PBS) as the swelling medium, rather than pure water, as more biologically relevant.^6^

DSC has been used to characterize bound and free water populations, with free water capable of freezing and tightly bound water unable to freeze.^7−9^ Antonsen and Hoffman studied various molecular weight PEGs using DSC and found that the number of bound water molecules per repeat unit ranged from 2.3 to 3.8.^10^ Li et al. used DSC measurements to determine how the fraction of free water in poly(vinyl alcohol) (PVA) hydrogels changed with samples that had been swollen to varying degrees.^8^ Above a critical threshold, the fraction of free water in the gel increased linearly with the degree of saturation or extent of swelling. Multiple endothermic peaks were also observed in DSC scans, suggesting multiple states of freezable water.^8^ This other type of freezable water, which freezes below 0 °C, is known as intermediate water and is considered to be a type of bound water.^11^ The presence of these water molecules on the polymeric surface can prevent high-molecular-weight molecules, such as plasma proteins, from adsorbing, and thus can provide hemocompatibility.^11^

Evidence of more than two states of water has also been reported by Yang et al.; from DSC results, up to six bound water molecules were calculated per PEG unit in PEGDA hydrogels, suggesting that nonfreezable water molecules can also represent those not directly bound to the polymer chain.^12^ Ahmad and Huglin used DSC to determine the states of water present in poly(methyl methacrylate-*co*-*N*-vinyl-2-pyrrolidone) hydrogels.^9^ With an increasing concentration of *N*-vinyl-2-pyrrolidone (VP) units present in the dry gels, the binding ratio (ratio of bound water molecules to VP units) increased, ranging from 3.8 at lower concentrations to 7.5 at higher concentrations. Multiple endothermic peaks were also observed, suggesting intermediate populations of water.^9^

Vigata et al. found that gelatin methacroyl (GelMA) hydrogels swelled with PBS showed higher amounts of nonfreezable water than those swelled with water.^13^ This is likely due to the presence of ions, which can disrupt the organization of water when hydration shells are formed, likely leading to changes in the states of water present. Yang et al. observed similar results in PEGDA-based hydrogels, with hydrogels swelled in PBS exhibiting higher amounts of nonfreezable water than those swelled in water.^14^

NMR diffusion and relaxation measurements can also be used to study the behavior of water within hydrogels and be used to find tortuosity.^15−19^ Pulsed field gradient (PFG) NMR techniques can quantify water mobility from measurements of the self-diffusion of water within a sample. Barbieri et al. used NMR diffusion measurements to study water in poly(HEMA) and poly(HEMA-*co*-DHPMA) hydrogels. Using a Stejskal–Tanner PFGSE technique, a single diffusion coefficient was measured for the population of water within the hydrogels. The measured diffusion coefficient increased with increasing water fraction in the hydrogels.^15^

McConville and Pope performed diffusion measurements on different contact lens hydrogels and found an increase in the measured diffusion coefficient with increasing equilibrium water content (EWC) of the different hydrogel materials.^16^ The ratio of bound water to dry polymer also increased with EWC. Each of the hydrogel materials that they studied was dried to different extents, such that the water content of each material was no longer at EWC. It was found that the fraction of water molecules that were bound to the polymer decreased as water content increased for a particular hydrogel.^16^

Multiple factors can impact the mobility of water molecules within a hydrogel. The water may be nearly immobilized through its chemical interaction with the polymer chains (the bound state), or it may be free to move within the polymer network restricted only by the network formed by the polymer. Measurements of relaxation times and diffusion coefficients in such settings are exchange-rate averages of the two states. In the fast-exchange limit, a single value for either the relaxation time or diffusion coefficient will be observed. In this study, the fast-exchange limit will allow changes in diffusion coefficients with water content within the ETTMP–PEGDA hydrogels to be related to the fractions of free and bound water in the system.

## 2 Materials and Methods

### 2.1 Materials

PEGDA (*M*_n_ = 575 g/mol) was purchased from Sigma-Aldrich (St. Louis, Missouri), purified, filtered, and stored at 4 °C. ETTMP (THIOCURE ETTMP 1300; *M*_n_ = 1300 g/mol) was purchased from Bruno Bock (Marschacht, Germany) and purified, filtered, and stored at 4 °C. Brockmann I basic aluminum oxide was purchased from Sigma-Aldrich (St. Louis, Missouri).

### 2.2 Hydrogel Preparation

ETTMP and PEGDA are purified using a 3.8 cm column of basic aluminum oxide to remove the radical inhibitor hydroquinone monomethyl ether (MEHQ) from PEGDA and degraded mercaptopropionic acid from ETTMP, which are further purified using a 0.45 μm syringe filter (Fisher Scientific, Waltham, Massachusetts) to remove any remaining alumina particles. The ETTMP and PEGDA are added to a vial in a 2:3 stoichiometric ratio and vortexed for 15 s (Mini Vortex Mixer, VWR, Radnor, Pennsylvania). PBS solution (0.1 M, pH 7.40) is then added to the vial to create the desired hydrogel formulation. The mixture is then vortexed for 15 s, yielding a clear solution that forms a clear hydrogel. Polymer mass fractions around 0.15 begin to approach the limit of gel formation.

### 2.3 DSC Testing of Hydrogels

Hydrogels are prepared as described above and pipetted into a silicone mold to prepare a 1 mm thick hydrogel disk. The hydrogels are allowed to cure in a 37 °C oven for 20 min, after which the gel disks are cut into small pieces (3 to 6 mg) and sealed in TZero aluminum pans with a hermetic lid (TA Instruments, New Castle, Delaware), before being placed into the sample tray of a Q2000 DSC (TA Instruments, New Castle, Delaware). Before each hydrogel sample run, a sample of deionized (DI) H_2_O is run. The melting peak associated with the DI H_2_O is taken as the latent heat of pure water and is used in calculations. Samples are cooled to −40 °C at a rate of 2 °C/min, equilibrated at −40 °C, and heated to 40 °C at a rate of 2 °C/min to observe the melting peak of free water. From these measurements, the mass fraction of free water in the gel, , can be calculated from
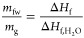
1where *m*_fw_ is the mass of free water, *m*_g_ is the mass of gel,  is the enthalpy of fusion of pure water, and Δ*H*_f_ is the enthalpy of fusion of water in the hydrogel observed for each sample.^8^ The mass fraction of water that is free is found via
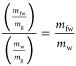
2where  is the mass fraction of water in the gel, known from hydrogel composition. The mass fraction of water that is bound, , is then
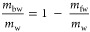
3The mass fraction of bound water to polymer, , can be found, using the polymer mass fraction, 
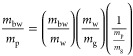
4

### 2.4 NMR Diffusion Measurements

Vortexed hydrogel solutions are pipetted into NMR tubes (Wilmad-LabGlass, Vineland, New Jersey) and allowed to cure in a 37 °C oven for 20 min. The samples are kept at 25 °C until measurements are made. Diffusion measurements were made at 25, 30, and 37 °C. The samples are allowed to equilibrate in the instrument (600 MHz Varian NMR spectrometer, Agilent Technologies, Santa Clara, California) at 25 °C for 10 min and at 30 and 37 °C for 1 h before measurements at each respective temperature are started. For each temperature, the π/2 pulse time is determined before making measurements. A stimulated spin-echo pulse sequence, based on the sequence described by Wu et al., was used for diffusion coefficient measurements.^20,21^ The expected signal for this measurement is a function of the Stejskal–Tanner variable, *X*, defined in terms of the pulse–sequence parameters as
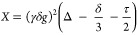
5Experiments used gradient-pulse amplitudes, *g*, that varied from 0 to 61.5 G/cm with a fixed gradient-pulse duration (δ = 2 ms) and gradient-pulse separation time (Δ = 100 ms). The variable-time delay, τ, was computed to ensure proper timings for a measurement and was typically 1.5 ms. A diffusion coefficient, *D*, can then be calculated using
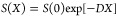
6where *S*(*X*) is the signal for a given value of *X* and *S*(0) is the signal without a gradient pulse. The signal is taken from the water peak in the ^1^H NMR spectrum, around 4.0 ppm.

### 2.5 NMR Relaxation Measurements

Hydrogel samples were prepared as described for the NMR diffusion measurements and were measured at 25 °C using the same instrumentation. As with the diffusion measurements, the π/2 pulse time was determined before each run. A Carr–Purcell–Meiboom–Gill pulse sequence was used for finding spectrally resolved *T*_2_ relaxation times, with the separation between pulses, τ, set to 0.1 ms.

### 2.6 Equilibrium Swelling Content Studies

Hydrogel tablets are made by pipetting 100 μL of hydrogel solution into a Delran mold to create the 3 mm thick tablet with 6 mm diameter. The tablets are cured in a 37 °C oven for 20 min. The tablets are massed and placed in 45 mL of pH 7.4 0.1 M PBS in a 50 mL centrifuge tube (VWR, Radnor, Pennsylvania) and allowed to reach equilibrium at the set temperature while agitated at 125 rpm for 24 h. Separate samples were equilibrated at 25, 30, and 37 °C to determine EWC at different temperatures. After 24 h, the hydrogel tablets are removed, patted dry, and massed to obtain the mass of the hydrogel after equilibration. The tablets are then lyophilized (4.5 L, −105 °C, equipped with a small tray dryer, Labconco, Kansas City, Missouri) for 12 h to obtain the dry polymer mass.

## 3 Results and Discussion

### 3.1 Differential Scanning Calorimetry

**Figure 1:**
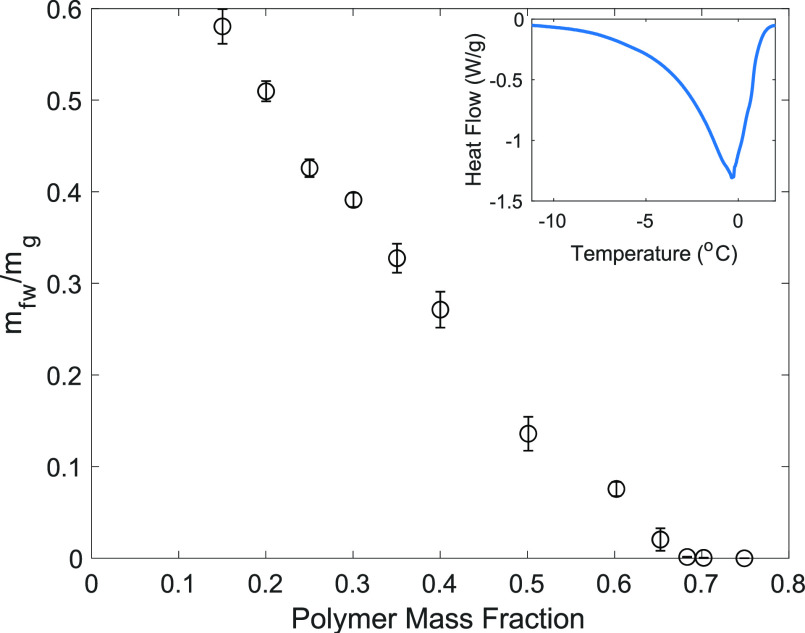
Mass of free water present per mass of gel  in hydrogels of varying polymer mass fractions, obtained with DSC measurements of the freezing peaks of water in hydrogels of varying polymer mass fractions (*n* = 3; ±standard error). Inset: DSC curve for the melting peak of water in the hydrogel at a polymer mass fraction of 0.25.

The DSC thermograms show an endothermic peak at or around 0 °C, associated with the freezable water, for all compositions of the hydrogel. It is possible that the melting of two different species of water is present, but the resolution is such that the close peaks of freezable water (free and intermediate) cannot be differentiated. Because of this, the freezable water is referred to as free water, with the understanding that some portion of this water is likely loosely bound or intermediate water. The DSC results showed a decrease in the mass of free water per mass of gel with an increasing polymer mass fraction of the hydrogel (Figure 1). At polymer mass fractions above 0.68, there is no measurable freezable water, according to the DSC results, and all remaining water in the sample is assumed to be tightly bound or nonfreezable water. As a hydrogel swells and absorbs more water, polar regions of the hydrogel are first hydrated; as the chains become saturated, water begins to fill the network openings. These two processes account for the changing fraction of bound, nonfreezable water molecules as the hydrogel swells to a greater extent.

### 3.2 NMR Diffusion and Relaxation Measurements

**Figure 2:**
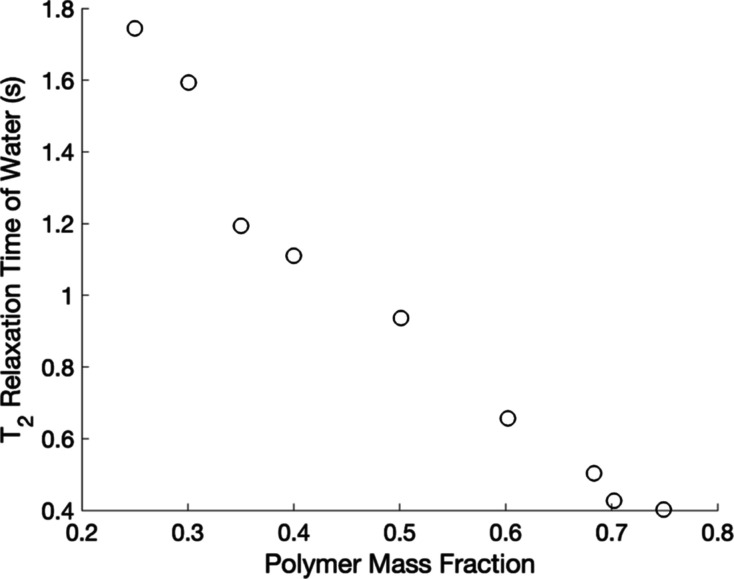
Spectrally resolved *T*_2_ relaxation times of water in hydrogels of varying polymer mass, as measured via NMR at 25 °C.

Since both freezable (free) and nonfreezable (bound) water were observed in this system, it was expected that NMR relaxation and diffusion measurements would yield signals that represented both populations. Spectrally resolved *T*_2_ measurements yielded only one observable relaxation time for the water protons, representing an average value resulting from the fast exchange between free and bound water populations. The *T*_2_ relaxation rate measured decreased with decreasing water in the hydrogel, as shown in Figure 2. Although the intrinsic relaxation rate of each population should not change with concentration, the exchange between the populations, whose concentrations relative to each other are changing, affects the observed *T*_2_ value. Similarly, the diffusion measurements should show a single exchange-averaged value, and thus, eq 6 could be used to extract a single diffusion coefficient.

**Figure 3:**
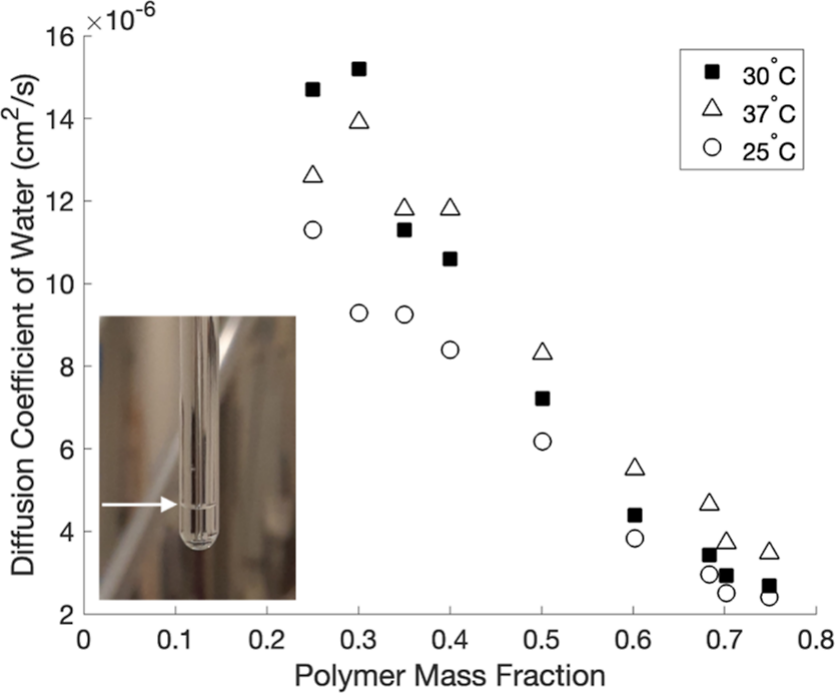
Diffusion coefficients of water measured via NMR at 25, 30, and 37 °C for hydrogels of varying polymer mass fraction. The values of 0.30 and 0.40 are the averages of two samples. The inset image shows the NMR tube of the clear hydrogel at a polymer mass fraction of 0.30 above a layer of expelled water, after measurements at 37 °C; the arrow indicates the interface between expelled water and hydrogel.

The changes in the observed diffusion coefficient, shown in Figure 3, as well as the trend noted in *T*_2_ relaxation rates, suggest that fast exchange between an essentially unobservable, low mobility population of water molecules associated with the polymer fraction and an observable more mobile population in the network openings can describe the trends shown here. Hence, we interpret the observed diffusion coefficient as the weighted average of the diffusion coefficients of free and bound water populations.

Since DSC measurements showed that only bound water was present in hydrogels at polymer mass fractions of 0.68 and greater, the diffusion measurements at a polymer mass fraction of 0.75 were assumed to essentially reflect the diffusion coefficient of bound water, *D*_bw_, at each temperature. Using these bound-water values, the observed value of the diffusion coefficient, *D*, is given by
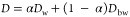
7In this expression, *D* is the diffusion coefficient obtained from the fit of eq 6 to the PFG measurements, *D*_w_ is the diffusion coefficient of pure water (Table 1), from the literature, and α is the fraction of water in the sample that is free.^22^ Rearranging eq 7 then provides a value for α as
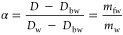
8

**Table 1:** Diffusion Coefficients of Water Measured by Holz et al.

temperature (°C)	experimental diffusion coefficients for water (× 10^–5^ cm^2^/s)^22^
25	2.299
30	2.597
37^a^	3.100

a Value at 37 °C extrapolated.

**Figure 4:**
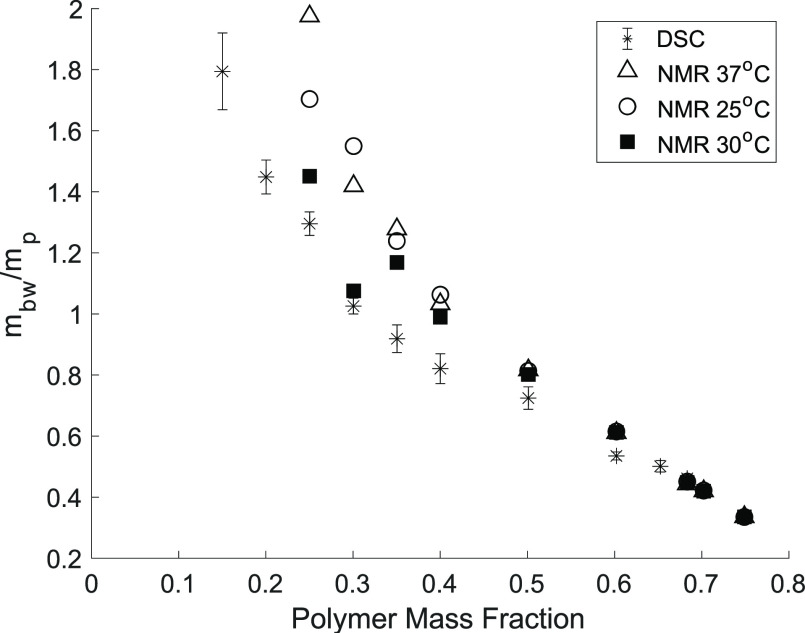
Comparison of the mass of bound water present per mass of the polymer  obtained via NMR (diffusion) and DSC (freezing; *n* = 3; ±standard error) measurements for said hydrogels.

Using eq 4, the NMR diffusion measurements can provide the mass fraction of bound water per polymer, , as a function of the polymer mass fraction for the sample. Figure 4 shows the comparison of the NMR results with the DSC measurements, calculated from eq 4, demonstrating good correspondence between the two approaches to the bound water measurement.

The DSC and NMR results are in good agreement, especially for hydrogels of higher polymer mass fraction. Small differences in the fraction of bound water are expected due to the temperature differences between the techniques and how these fractions are calculated. It is likely that the bound water ratios found via DSC would change in the ETTMP/PEGDA hydrogel if DI H_2_O was used instead of PBS in gel formation, as has been reported for other hydrogels.^13,14^ However, the gelation kinetics are favorable at higher pH in the ETTMP/PEGDA hydrogels, so PBS is used as the solvent here. These ionic interactions likely detected in DSC may not be as prevalent in NMR diffusion measurements and could lead to discrepancies between the two.

### 3.3 Equilibrium Water Content

**Figure 5:**
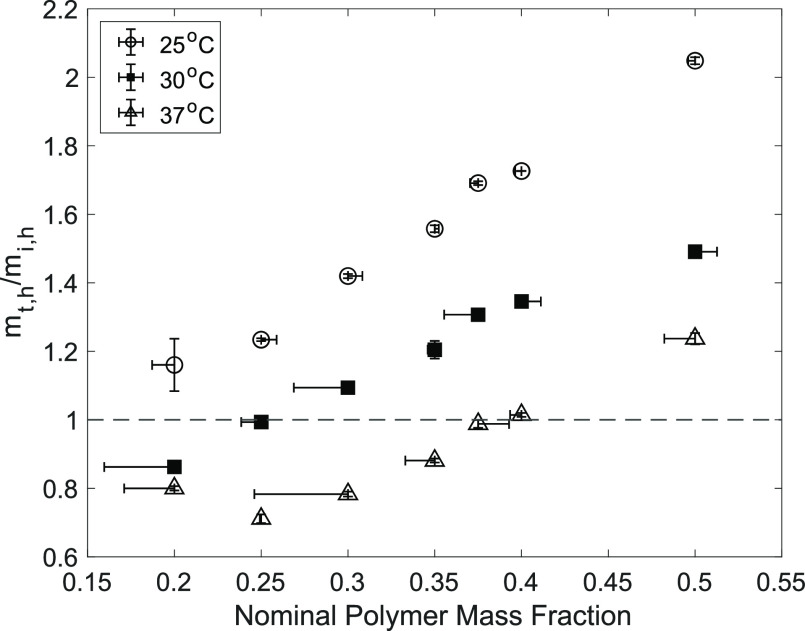
Fraction of initial mass of hydrated hydrogels, , present after 24 h in PBS at 25, 30, and 37 °C. (*n* = 3; vertical error bars represent ± standard error; horizontal error bars extend to the average polymer mass fraction obtained from lyophilized hydrogel mass (v. nominal masses as prepared); and the dotted line indicates EWC).

To determine EWC at 25, 30, and 37 °C, hydrogels at polymer mass fractions of 0.20 to 0.75 were allowed to swell or deswell for 24 h. Masses were taken of the hydrated hydrogel (polymer and water) before and after equilibration and of the dry hydrogel (polymer) following lyophilization. The initial mass of the hydrogels as prepared, *m*_i,h_, is taken before equilibration in PBS. The mass of the hydrogels after 24 h, *m*_t,h_, is taken such that the fraction of initial hydrated mass present after 24 h, , can be calculated, and this value is plotted against polymer mass fraction at each temperature in Figure 5. Mass loss, represented by values of  less than 1, is seen in hydrogel compositions with water content greater than EWC at a given temperature, due to deswelling and loss of water. Hydrogel compositions with water content less than EWC swell, demonstrating a gain in mass and  values greater than 1. At 30 and 37 °C, the compositions at equilibrium, represented by a  value of 1, are 0.25 and 0.375 polymer mass fraction, respectively. Overall, a lower extent of swelling is seen at higher temperatures for all compositions. Some deviations in polymer mass fraction obtained after lyophilization are seen and can be attributed to errors in obtaining mass, diffusion of the unreacted polymer from the hydrogel, and remaining water after lyophilization.

## 4 Conclusions

The amounts of freezable and nonfreezable water were characterized via DSC in ETTMP/PEGDA hydrogels of varying polymer mass fractions, and it was found that the mass fraction of nonfreezable water was relative to the polymer mass fraction and decreased with increasing polymer mass fraction. The NMR diffusion measurements yielded similar ratios. Diffusion coefficients for water generally increased with increasing temperature for each composition, except for hydrogels with a polymer mass fraction of 0.25 and 0.30, which are above EWC at 37 °C. Bound water fractions were obtained from the diffusion measurements by assuming that the diffusion coefficient measured was a weighted average of free and bound water. Understanding the states of water present in these hydrogels of varying composition is important to help us determine which composition may be needed for a certain application, as the presence of certain water states may have an impact on hemocompatibility.

Determining the extent of swelling and deswelling of different hydrogel formulations provides insights into the EWC of the hydrogels at different temperatures. The EWC data demonstrate the need to perform studies on injectable hydrogels at physiological temperatures to translate into how the hydrogel behaves in vivo. It is important to understand the swelling and deswelling and associated volume change of the hydrogel, since placement near sensitive tissues requires a hydrogel that maintains its initial volume and shrinks as degradation proceeds, such that pressure is not placed on the surrounding tissue. The implant should not, however, significantly shrink and migrate prior to releasing its payload. Initial swelling or deswelling of a hydrogel may also lead to changes in the drug release and degradation behaviors of the hydrogels; for example, a significant deswelling of a drug-loaded hydrogel may be accompanied by an initial burst of a drug. Future work is underway to understand how the hydrogel water content affects erosion and drug release.
